# GSK-3β phosphorylation-dependent degradation of ZNF281 by β-TrCP2 suppresses colorectal cancer progression

**DOI:** 10.18632/oncotarget.20100

**Published:** 2017-08-09

**Authors:** Yuekun Zhu, Qingxin Zhou, Guiling Zhu, Yanwei Xing, Shiqiang Li, Niansheng Ren, Tianyou Liu, Anlong Zhu, Yuxian Bai, Daxun Piao

**Affiliations:** ^1^ Department of Colorectal Surgery, The First Affiliated Hospital of Harbin Medical University, Harbin 150001, Heilongjiang Province, China; ^2^ Department of Gastrointestinal Oncology, Harbin Medical University Cancer Hospital, Harbin 150086, Heilongjiang Province, China

**Keywords:** colorectal cancer, zinc finger protein 281, the β-transducin repeat-containing protein 2, glycogen synthase kinase 3β, ubiquitination

## Abstract

Zinc finger protein 281 (ZNF281) has been recently shown to be critical for CRC progression. However, the immediate upstream regulators of ZNF281 remain unclear. Here we reported that the E3 ligase the β-transducin repeat-containing protein 2 (β-TrCP2) governs the ubiquitination and degradation of ZNF281. In human CRC specimens, endogenous β-TrCP2 were inversely correlated with ZNF281. Beta-TrCP2 reversed the phenotype of CRC cell with overexpressed ZNF281. Moreover, we found that glycogen synthase kinase 3β (GSK-3β), not GSK-α, could bind to and phosphorylate ZNF281 at one consensus motif (TSGEHS; phosphorylation site is shown in italics), which promotes the interaction of ZNF281 with β-TrCP2, not β-TrCP1, and leads to the subsequent ubiquitination and degradation of phosphorylated ZNF281. A mutant of ZNF281 (ZNF281-S638A) is much more stable than wild-type ZNF281 because ZNF281-S638A mutant abolishes the phosphorylation by GSK-3β and can not be ubiquitinated and degraded by β-TrCP2. Conversely, ZNF281 transcriptionally repressed the expression of β-TrCP2, indicating a negative feedback loop between ZNF281 and β-TrCP2 in CRC cells. These findings suggest that the turnover of ZNF281 by β-TrCP2 might provide a potentially novel treatment for patients with CRC.

## INTRODUCTION

Colorectal cancer (CRC) is the third-leading causes of death, and surgical resection is the standard treatment for early stage CRC [1]. Annually CRC accounts for about 10% of all cancer cases with more than a million new cases diagnosed worldwide [2]. Mainly due to improvements in care in recent decades and the introduction of multidisciplinary treatment, the oncological outcome of CRC has greatly improved [3]. However, 30%–40% of these patients will experience the recurrence of the disease. Widespread metastasis is the main cause of cancer-related death [4]. Up to now, efforts to prevent metastasis and increase cure rates after surgery have focused on combined chemotherapy administration [5]. Unfortunately, such therapy is unsatisfactory in reducing metastatic relapse. Growing evidence suggests that transcription factors regulating gene expression and certain signaling pathways during carcinogenesis are potential therapeutic targets because they control tumor initiation and progression [6]. But the signaling pathways controlling the expression of these important transcription factors are not yet understood.

ZNF281, also known as ZBP-99 or ZNP-99, has first been identified as a 99 kDa Krüppel-type zinc-finger transcriptional regulator [7]. Given that ZNF281 binds to GC-rich regions located in the promoters of a variety of genes, it was first named GC-box-binding zinc-finger protein (GZP1) [8]. The ZNF281 gene is phylogenetically conserved and located on chromosome 1q32.1 [9]. Recently, ZNF281 has been characterized as an epithelial-mesenchymal transition (EMT)-inducing transcription factor (EMT-TF), suggesting that ZNF281 may have a crucial role in controlling migration, invasion, and metastasis in tumors [10]. More recently, ZNF281 was recognized as a directly transcriptional repressor of Nanog [11]. Therefore ZNF281 was regarded as a relevant gene for cellular stemness [12]. On the other hand, the EMT-TF SNAIL and SOX4 were demonstrated to directly induce the transcription of ZNF281 [13]. And p53 posttranscriptionally inhibited the expression of ZNF281 through a miR-34a-dependent mechanism [13]. Although DNA damage leads to the phosphorylation of ZNF281 protein by ataxia telangiectasia-mutated (ATM) and ataxia telangiectasia-mutated- and Rad3-related (ATR) kinases [14], other posttranslational modification of ZNF281 remains to be understood in greater detail. Given that high ZNF281 expression in CRC significantly are correlated with the tumor stage, and that ZNF281 related biology has the potential to be translated into cancer diagnostic, prognostic, and therapeutic methods [15], thus, we are interested in investigating how the level of ZNF281 is regulated in the tumorigenesis of CRC.

In the present study, we investigated the regulatory mechanisms of ZNF281 protein expression in human CRC. We show that GSK-3β physically associated with and phosphorylated ZNF281, and that the phosphorylated ZNF281 was then selectively ubiquitinated and degraded by the E3 ligase β-TrCP2, not β-TrCP1. A ZNF281 mutant (ZNF281-S638A), which cannot be degraded by β-TrCP2, enhanced CRC growth and metastasis. ZNF281, in turn, repressed the transcription of β-TrCP2 in CRC. These findings will potentially serve as a basis for novel CRC diagnostic and therapeutic approaches in the future.

## RESULTS

### The inverse expression of ZNF281 and β-TrCP2 in human CRC tissues are correlated with clinical outcome

**Figure 1:**
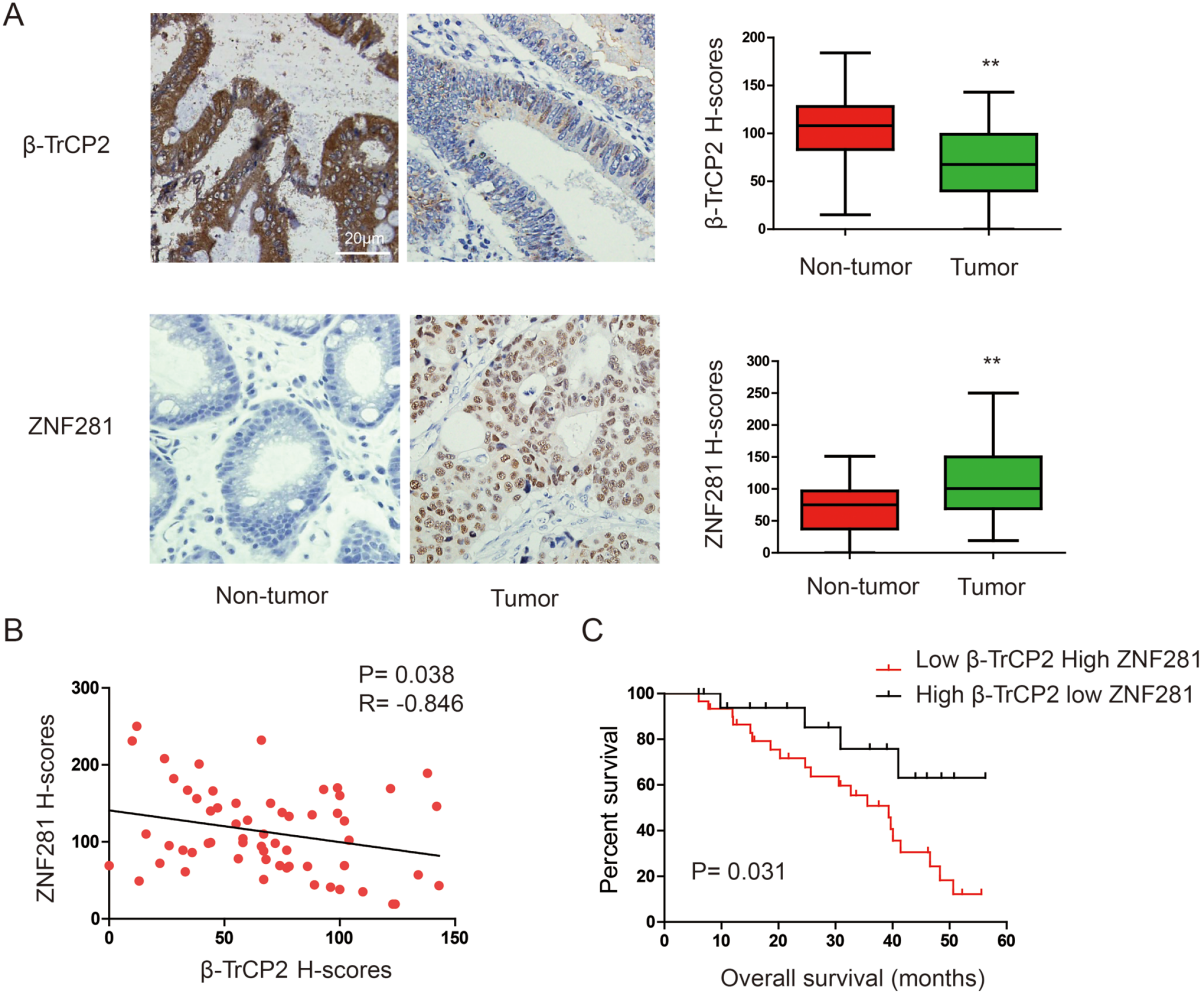
Expression patterns of β-TrCP2 and ZNF281 in human CRC **(A)** representative images of IHC staining of β-TrCP2 and ZNF281 conducted in CRC tissues and paired non-tumor tissues (left panel). The expression was quantified by H-scores (right panel). **(B)** Linear correlation analysis was used to detect the correlationship between β-TrCP2 and NF90 expression. **(C)** Kaplan-Meier curves show that HCC patients with high ZNF281 and low β-TrCP2 H-scores had a poorer prognosis than patients with low ZNF281 and high β-TrCP2 H-scores did. **P < 0.01.

ZNF281 has been suggested to play a pivotal role in CRC [15]. However, the mechanism by which the level of ZNF281 protein was regulated in CRC remains to be elucidated. Given that ZNF281 contains a sequence, TSGXXS, which is similar to the DSGXXS/T destruction motif in known substrates of the E3 ligase β-TrCP2 [16], we reasoned that β-TrCP2 might be an upstream regulator of ZNF281. To test this hypothesis, we firstly investigated the expression patterns of β-TrCP2 and ZNF281 in human 60 CRC tissues and corresponding adjacent non-tumor tissues. Results of immunohistochemical (IHC) analysis showed that ZNF281 levels in CRC samples were significantly higher compared with adjacent non-cancerous tissues. In contrast, β-TrCP2 levels in tumors were significantly lower compared with adjacent non-tumor tissues (Figure 1A). These data suggested that ZNF281 upregulation correlated with β-TrCP2 downregulation in CRC (Supplementary Table 1). Indeed, linear correlation analysis showed an inverse correlation between ZNF281 and β-TrCP2 (R = -0.846, P = 0.0.38, Figure 1B). We further investigated the effect of ZNF281 and β-TrCP2 expression pattern on the prognosis of CRC patients. The Kaplan-Meier survival analysis suggested that expression of high ZNF281 with concurrent low β-TrCP2 tended to correlate with poor prognosis (Figure 1C).

### β-TrCP2, not β-TrCP1 overexpression inhibits the effects of ZNF281 in CRC *in vitro* and *in vivo*

**Figure 2:**
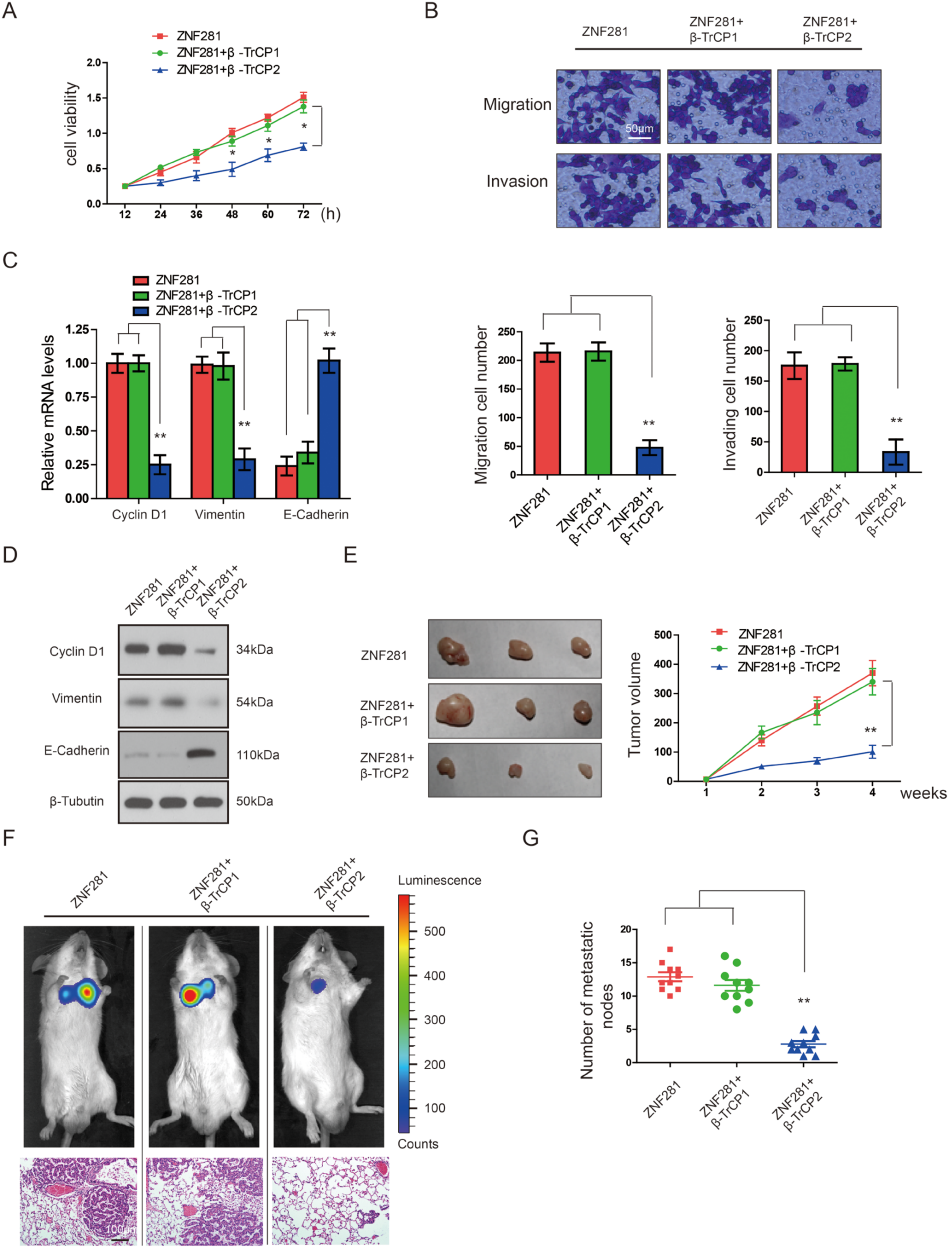
β-TrCP2, not β-TrCP1 overexpression inhibits the effects of ZNF281 in CRC *in vitro* and *in vivo* HCT116 cells were transfected with ZNF281 alone, plus β-TrCP1, or β-TrCP2 respectively. **(A)** Cell proliferation experiment was performed using CCK-8 assay. **(B)** Cell migration and invasion by Transwell assay was performed. **(C)** and **(D)** The mRNA and protein expression of the EMT-related markers Vimentin, Cyclin D, and E-cadherin examined by qRT-PCR and western blotting. **(E)** Indicated HCT116 cells were inoculated subcutaneously into the right flank of male SCID mice (n = 9 for each group). Tumor volume measured with a caliper. **(F)** Representative images of *in vivo* imaging analysis indicates lung metastatic nodules. **(G)** The number of lung visible metastatic nodules was shown in HCT116 cells with indicated treatment. **P <0.01.

Next, we test whether β-TrCP2 affects the functions of ZNF281 in CRC. β-TrCP2 and its isoform β-TrCP1 belong to β-TrCP family [17]. In order to confirm that the effect of β-TrCP2 on the functions of ZNF281 is unique, we also test whether β-TrCP1 affects the functions of ZNF281 in CRC. Consistent with a previous report [15], overexpression of ZNF281 indeed promoted the proliferation, migration and invasion of CRC cells while transfection of β-TrCP2, not β-TrCP1, significantly inhibited ZNF281-induced CRC cell proliferation, migration and invasion (Figure 2A and 2B), suggesting that only β-TrCP2 is an upstream regulator of ZNF281. The above data was further evidenced by increased expression of the epithelial marker E-cadherin and decreased expression of the mesenchymal marker Vimentin and Cyclin D1 induced by β-TrCP2 (Figure 2C and 2D). These data strongly demonstrated that β-TrCP2 decreased ZNF281-induced CRC cell growth, migration, and EMT *in vitro*. In contrast, β-TrCP2 shRNA failed to rescue the phenotypes of ZNF281 in CRC cells (data not shown).

To clearly demonstrate whether our *in vitro* data can be repeated *in vivo*, we established a mouse CRC xenograft model by subcutaneously injecting the severe combined immunodeficient (SCID) mice with HCT116 cells with ZNF281 overexpression as a control. It was observed that mice from the HCT116 cells transfected with β-TrCP2 and ZNF281 showed significantly decelerated tumor growth (Figure 2E). To assess whether β-TrCP2 inhibits metastasis, we intravenously injected β-TrCP2-overexpression HCT116 cells, with ZNF281 overexpression as a control, into SCID mice and subjected to bioluminescent imaging. β-TrCP2-overexpression cells exhibited reduced number of lung metastasis compared with control (Figure 2F and 2G). These results demonstrated that β-TrCP2 inhibited CRC tumorigenesis and metastasis *in vivo*. Thus, ZNF281 inhibition by β-TrCP2 might provide therapeutic benefits for CRC.

### Disruption of the β-TrCP2, not β-TrCP1 gene, induces accumulation of ZNF281 in MEF cells

**Figure 3:**
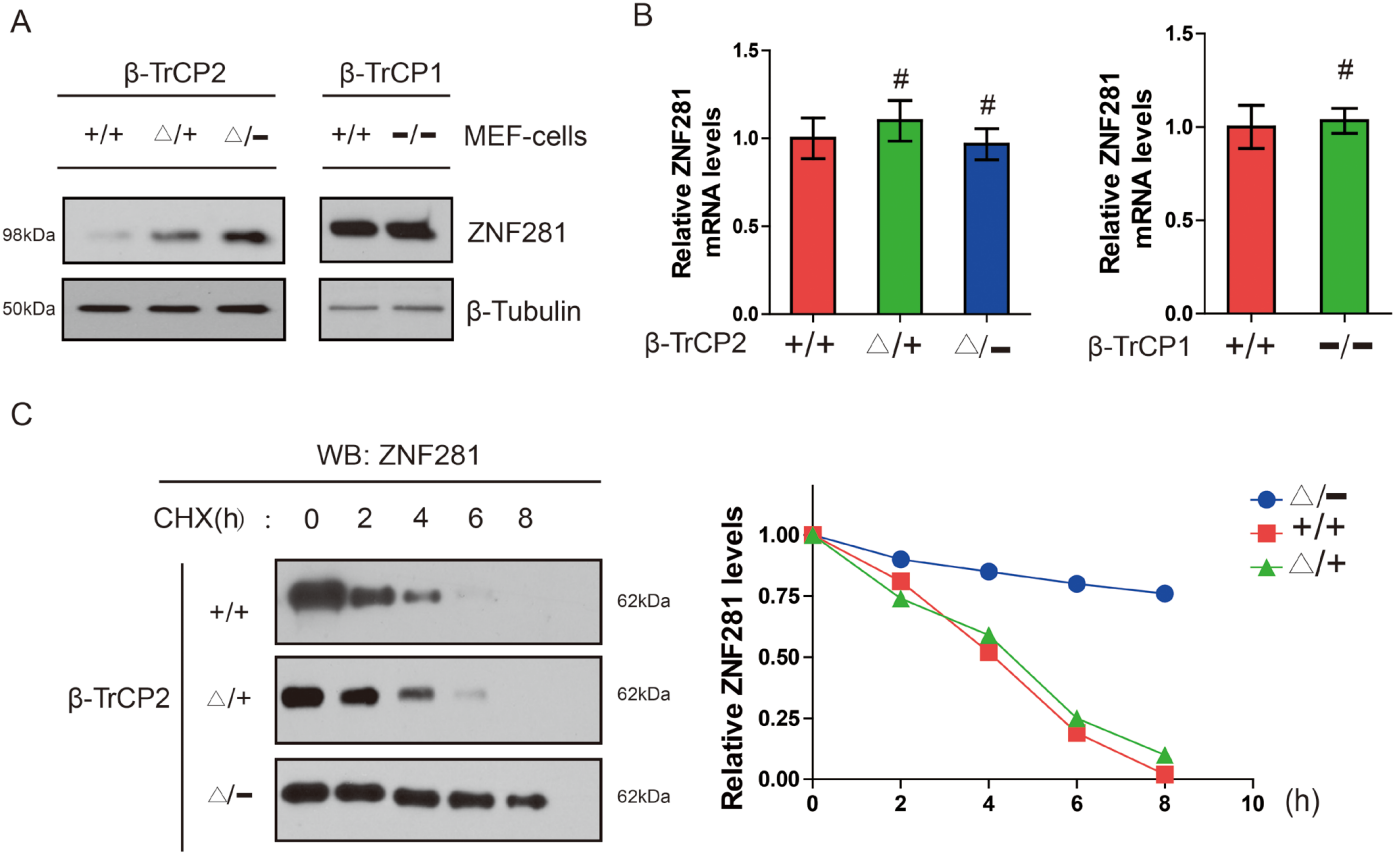
Disruption of the β-TrCP2, not β-TrCP1 gene, induces accumulation of ZNF281 in MEF cells **(A)** Measurement of ZNF281 protein level in MEFs of the indicated β-TrCP2 and β-TrCP1 genotypes. Tubulin served as a loading control. **(B)** Measurement of ZNF281 mRNA levle in MEFs of the indicated β-TrCP2 and β-TrCP1 genotypes using RT-qPCR. **(C)** Measurement of ZNF281 in MEFs of the indicated genotypes treated with CHX (25 μg/ml) for the indicated times. And relative ZNF281 abundance normalized by tubulin abundance.

Given that β-TrCP2 is an E3 ligase and ZNF281 contains a β-TrCP degron sequence, we focused on ZNF281 as a potential novel substrate of β-TrCP2. Thus, we hypothesized that β-TrCP2 affects the stabilization of ZNF281 and loss of β-TrCP2 would be expected to induce accumulation of its ZNF281. To characterize the molecular basis of the inhibition of ZNF281-induced proliferation and migration in CRC cells following β-TrCP2 overexpression, we, therefore, examined the abundances of ZNF281 in MEF cells with β-TrCP1 or β-TrCP2 knockout respectively. We found that the level of ZNF281 protein was upregulated in the β-TrCP2 knockout MEFs, while there was no substantial accumulation of ZNF281 in β-TrCP1Δ/− MEFs (Figure 3A). Moreover, the abundance of ZNF281 mRNA was not markedly increased in β-TrCP2Δ/− MEFs (Figure 3B), suggesting that ZNF281 accumulation did not result from transcriptional regulation. CHX chase analysis indicated that ZNF281 was stabilized in these mutant MEFs (Figure 3C). These results strongly support the notion that ZNF281 is a specific substrate of β-TrCP2, not β-TrCP1. Together, our data indicated that β-TrCP2, but not β-TrCP1, induces the degradation of ZNF281 in MEF cells.

### β-TrCP2 overexpression induced K48-linked ZNF281 ubiquitination while knockdown of β-TrCP2 had the opposite effect

**Figure 4:**
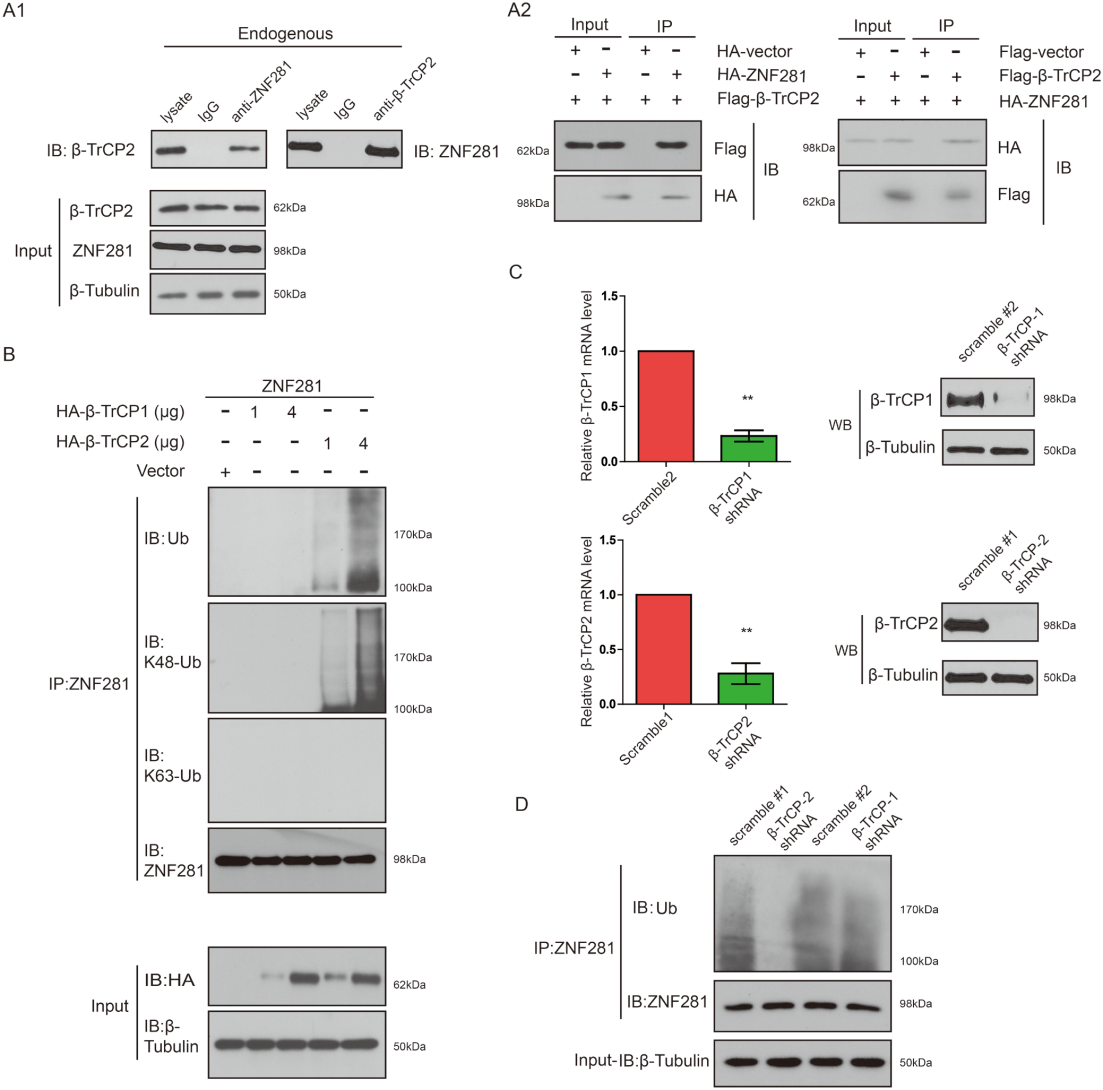
β-TrCP2 overexpression induced K48-linked ZNF281 ubiquitination while knockdown of β-TrCP2 had the opposite effect **(A1)** Endogenous proteins in total lysates of HCT116 cells were subjected to IP with indicated antibody followed by immunoblotting with anti-β-TrCP2 or anti-ZNF281 antibody. A rabbit IgG was included as an IP negative control. **(A2)** HCT116 cells were transfected with ZNF281 and β-TrCP2 constructs. The cell lysates were subjected to immunoprecipitation with indicated antibody and then analyzed by Western blotting. **(B)** HCT116 cells were transfected with the indicated amounts of expression vectors. Then cell lysates of indicated cells were subjected to immunoprecipitation with anti-ZNF281 antibody and were analyzed by Western blotting with anti-ubiquitin antibody, K48-specific ubiquitin antibody, and K63-specific ubiquitin antibody. **(C)** HEK293T cells were infected with lentiviral β-TrCP1 shRNA, or nontargeting shRNA, or lentiviral β-TrCP2 shRNA. The knockdown efficiency was analyzed by human β-TrCP1- or β-TrCP2-specific qRT-PCR or Western blotting. **(D)** Lysates of the β-TrCP1- or β-TrCP2 knockdown or control cells were subjected to immunoprecipitation with anti-ZNF281 and analyzed by Western blotting with antiubiquitin antibody.

To explore the ability of β-TrCP2 to interact with endogenous ZNF281, we carried out co-immunoprecipitation (co-IP) with an antibody against ZNF281 or a reciprocal co-IP with an antibody against β-TrCP2. As expected, we found that endogenous ZNF281 protein was associated with β-TrCP2 protein *in vivo* (Figure 4A1). Using the co-transfection method, we further confirmed that HA-ZNF281 protein specifically binds to Flag-β-TrCP2 protein in the transfected cells (Figure 4A2).

Next, we set out to investigate whether β-TrCP2 indeed is an E3 ligase of ZNF281. As shown in Figure 4B, overexpression of β-TrCP2, not β-TrCP1, promoted ZNF281 ubiquitination in a dose-dependent manner. Furthermore, the β-TrCP2-mediated ZNF281 polyubiquitination chain was indeed K48, but not K63 linked (Figure 4B).

To determine whether endogenous β-TrCP2, not β-TrCP1 is indeed critical for ZNF281 K48-linked ubiquitination, we infected HEK293T cells with lentiviral β-TrCP1, or β-TrCP2 shRNA, or control (scramble) shRNA and the impact of β-TrCP1 or β-TrCP2 knockdown on ZNF281 degradation was examined. By real-time PCR and Western analysis, we showed that endogenous β-TrCP1 and β-TrCP2 were effectively knocked down (Figure 4C). We found that knockdown of β-TrCP2, not β-TrCP1, significantly attenuated ZNF281 ubiquitination (Figure 4D), further confirming the role of β-TrCP2 in K48-linked ZNF281 ubiquitination and degradation in CRC cells.

### GSK-3β, not GSK-3α, downregulates ZNF281 in CRC cells

**Figure 5:**
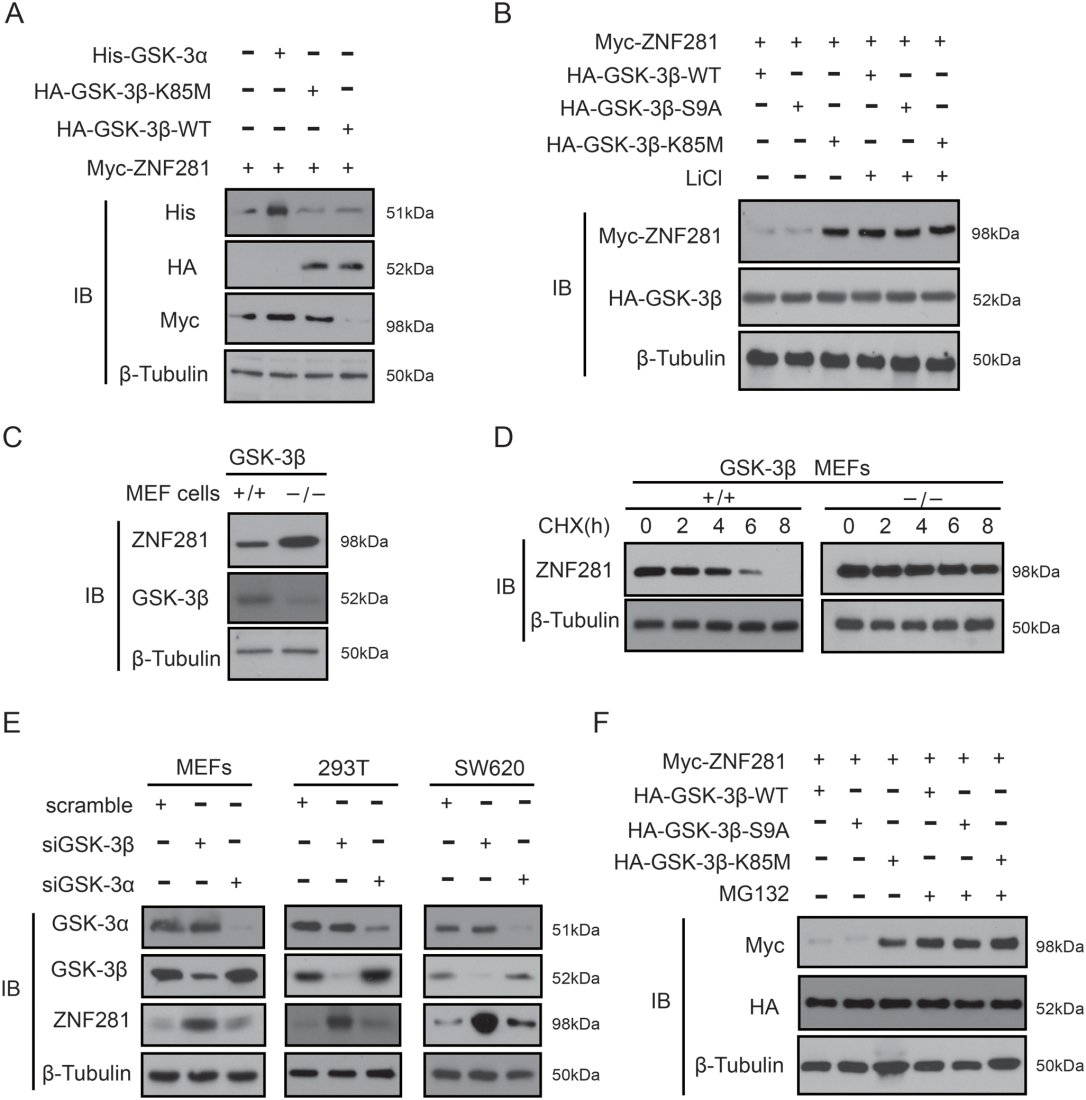
GSK--3β, not GSK-3α, decreases ZNF281 in CRC cells **(A** and **B)** HCT116 cells transfected with ZNF281, GSK-3β-WT, or GSK-3α, or GSK-3β-S9A, or GSK-3β-K85M were treated with or without the GSK-3β inhibitors LiCl (20 mM). Indicated proteins were then analyzed by Western blotting. **(C)** The whole lysates of wild type MEF cells and GSK-3β knockout MEF cells were subjected to immunoblotting with indicated antibodies. **(D)** CHX (20 μM) was used to treat MEF cells and GSK-3β knockout MEF cells for the indicated times, and endogenous ZNF281 was analyzed using western blot, and the half-lives of ZNF281 were determined. **(E)** siRNA against GSK-3β and GSK-3α was transfected into different kinds of human cell lines, and cell lysates were then analyzed to detect endogenous ZNF281. **(F)** HCT116 cells were transfected with indicated plasmids and treated with or without the proteasome inhibitor MG132 (10 μM) for 10 h. Then western blotting was performed.

It is well known that the β-TrCP degron must be phosphorylated to be recognized by β-TrCP1 or β-TrCP2 [18]. Thus, it is logical to examine whether β-TrCP2 binding to ZNF281 requires the phosphorylation of ZNF281. Given that the degron recognized by β-TrCP2 contains a conserved GSK-3 phosphorylation motif, we reasoned that GSK-3 possibly mediated the phosphorylation of ZNF281 which causes subsequent degradation of ZNF281. To confirm this idea, GSK-3β, or GSK-3α, or its mutant plus ZNF281 was transfected into HCT116 cells. It was found that ZNF281 expression levels were much lower in the presence of wild-type GSK-3β (GSK-3β-WT) than in the presence of kinase-dead GSK-3β (GSK-3β-K85M) and GSK-3α (Figure 5A). Furthermore, when the kinase activity of GSK-3β-WT or constitutively active GSK-3β (GSK-3β-S9A) was inhibited by a GSK-3β inhibitors lithium, levels of ZNF281 were increased (Figure 5B), indicating that GSK-3β, not GSK-3α, may decrease the protein level of ZNF281. The above results were further confirmed by our findings that the level of ZNF281 protein was significantly higher in the GSK-3β knockout MEF cells than that in WT MEF cells (Figure 5C), and that compared with WT-MEF cell, the half-life of endogenous ZNF281 in GSK-3β-knockout MEF cells increased from around 4 h to almost 6 to 8 h (Figure 5D). Moreover, silencing GSK-3β significantly increased the level of ZNF281 in different cell lines, while the knockdown of GSK-3α did not cause a similar result (Figure 5E). Interestingly, GSK-3β-S9A and GSK-3β-WT–induced ZNF281 decrease was significantly inhibited by the proteasome inhibitor MG132 (Figure 5F), suggesting that GSK-3β-induced ZNF281 downregulation is due to proteasome-mediated degradation.

### Phosphorylation of ZNF281 by GSK-3β is required for ZNF281 degradation

**Figure 6:**
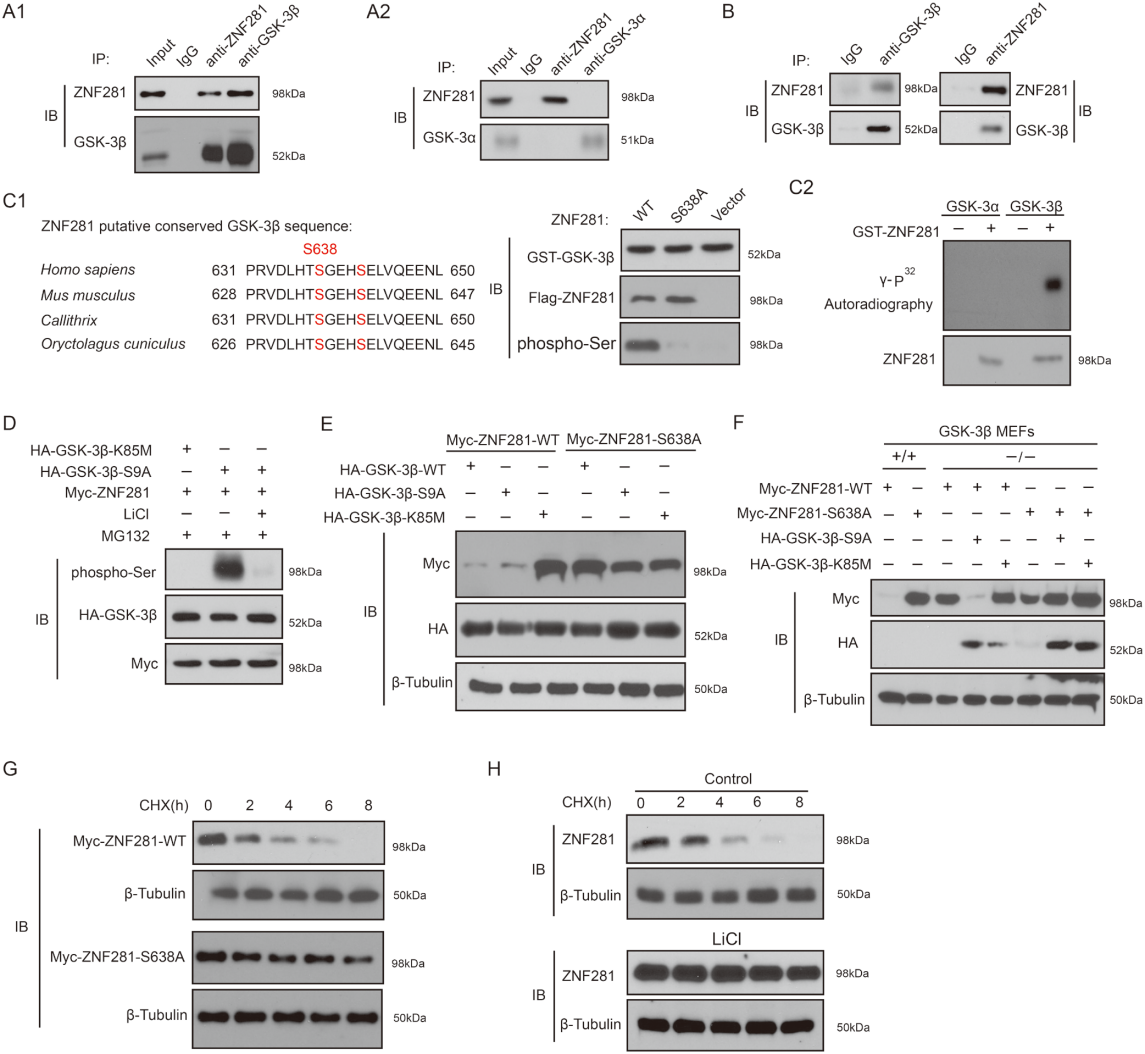
Phosphorylation of ZNF281 by GSK-3β is required for ZNF281 degradation **(A1** and **A2)** Co-transfection approach was used to investigate co-IP of GSK-3β with ZNF281. **(B)** Endogenous GSK-3β and ZNF281 were immunoprecipitated from HCT116 cells. (C) The amino acid sequences of the conserved GSK-3β phosphorylation motif in ZNF281 **(C1)**. GST-ZNF281 or its mutant protein was incubated with GST-GSK-3β, and the phosphorylation of ZNF281 was assayed with phosphor-Ser antibody (C1), and *in vitro* kinase assay was performed as described in Materials and Methods **(C2)**. **(D)** HCT116 cells were cotransfected with indicated plasmids, and then were treated with MG132 alone or with LiCl for 10 h. Samples were then blotted with specific phospho-Ser antibody. **(E)** WT-ZNF281 or its mutant was cotransfected with indicated plamids into HCT116 cells. Samples were then analyzed by Western blotting with indicated antibodies. **(F)** Indicated plasmids were cotransfected into WT-MEF or GSK-3β knockout MEF cells. Western blotting was performed with indicated antibodies. **(G)** Indicated plasmids were transfected into HCT116 cells, which were then treated with CHX (20 mM) for the indicated times. The levels of WT-ZNF281 and its mutant were determined. **(H)** HCT116 cells were treated with CHX (20 μM) with or without LiCl (20 mM) for the indicated times. Endogenous expression of ZNF281 was determined.

Next, we set out to demonstrate whether ZNF281 is a substrate of GSK-3. Co-transfection experiments indicated that GSK-3β interacts with ZNF281 while under the same condition, an interaction between ZNF281 and GSK-3α was not observed (Figure 6A1 and 6A2). We also detected the *in vivo* binding of endogenous ZNF281 to GSK-3β in HCT116 cells (Figure 6B). Given that β-TrCP2 degron of ZNF281 contains one conserved GSK-3 motif, thus whether GSK-3 could directly phosphorylate ZNF281 was investigated. As shown in Figure 6C, GSK-3β could phosphorylate ZNF281 while that Ser638 was mutated to produce ZNF281-S638A significantly abolished ZNF281 phosphorylation by GSK-3β (Figure 6C1). *In vitro* kinase assay further confirmed that ZNF281 is one direct substrate of GSK-3β, not GSK-3α (Figure 6C2). As expected, GSK-3β-mediated phosphorylation of ZNF281 was almost totally blocked by treatment with LiCl (Figure 6D). Taken together, these results showed that GSK-3β physically binds to ZNF281 and phosphorylates it at Ser638 site.

Next, the effect of ZNF281 phosphorylation at Ser638 site by GSK-3β on its stability was investigated. As shown in Figure 6E, compared to WT-ZNF281, ZNF281-S638A mutant was resistant to GSK-3β-S9A and GSK-3β-WT-induced decrease. In GSK-3β-WT-MEF cells, ZNF281-S638A mutant was also more stable than WT-ZNF281, while ZNF281-S638A mutant was as stable as WT-ZNF281 in GSK-3β knockout MEF cells, and the reintroduction of GSK-3β-S9A degraded WT-ZNF281, not ZNF281-S638A mutant (Figure 6F). Moreover, compared to the degradation rate of WT-ZNF281, the degradation rate of ZNF281-S638A mutant was significantly decreased (Figure 6G). The half-life of endogenous ZNF281 in HCT116 cells was delayed by the GSK-3β inhibitor lithium compared with that in cells without treatment (Figure 6H). Collectively, our data suggest that the phosphorylation of ZNF281 at S638 site by GSK-3β dictates its degradation.

### Phosphorylation of ZNF281 at Ser638 by GSK-3β promotes its binding to β-TrCP2 and degradation

**Figure 7:**
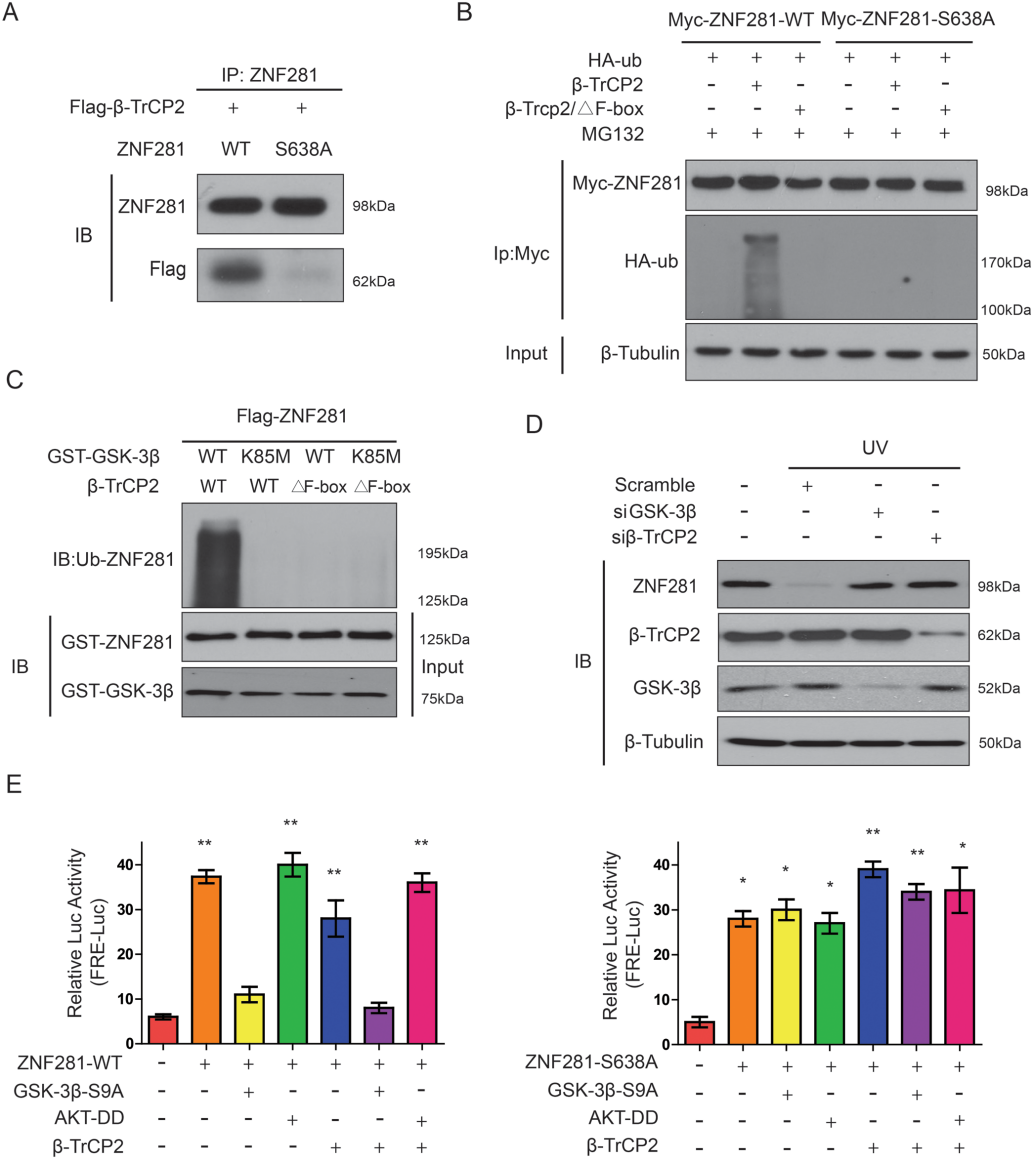
Phosphorylation of ZNF281 at Ser638 by GSK-3β promotes its binding to β-TrCP2 and degradation **(A)** Flag-β-TrCP2 was cotransfected with WT-ZNF281 or mutant ZNF281-S638A into HCT116 cells then treated with MG132 (10 μM) for 10 h. Flag-β-TrCP2 was immunoprecipitated and then analyzed for ZNF281. **(B)** HCT116 cells were transfected with indicated plasmids, and then treated with MG132 (10 μM). ZNF281 was immunoprecipitated and then analyzed with anti-HA-ubiquitin (HA-ub). **(C)** Purified ZNF281 protein was phosphorylated by GSK-3β and then incubated with *in vitro*-translated F-box-deleted β-TrCP2 and β-TrCP2 or in the presence of other indicated plasmids. ZNF281 ubiquitination was analyzed. **(D)** SiRNA against GSK-3β or β-TrCP2 were transfected for 48 h, and then HCT116 cells were stimulated with UV irradiation. ZNF281 phosphorylation was determined by Western blotting. **(E)** 293T cells cotransfected with firefly luciferase reporter containing ZNF281-responsive elements, pRL-TK as a transfection control for normalization), and other indicated plasmids, then samples were analyzed. *P <0.05, **P <0.01.

Next, we examined how GSK-3β-phosphorylated ZNF281 affected its ubiquitination and degradation by β-TrCP2 in CRC cells. Firstly, we examined whether GSK-3β-phosphorylated ZNF281 at Ser638 changed its binding to β-TrCP2. To this end, Flag-β-TrCP2 was cotransfected with WT-ZNF281 or ZNF281-S638A mutant into HCT116 cells, and cells were then treated with MG132. Co-IP assay indicated that the interaction between β-TrCP2 and WT-ZNF281 was obviously much stronger than the association between ZNF281-S638A and β-TrCP2 (Figure 7A), suggesting that the phosphorylation of ZNF281 at Ser638 by GSK-3β is required for its interaction with β-TrCP2. Then to further address whether β-TrCP2 directly ubiquitinated GSK-3β-phosphorylated ZNF281, an ubiquitination assay was performed. The results indicated that β-TrCP2 induced a much stronger ubiquitination in WT-ZNF281-expressing cells than in cells with ZNF281-S638A mutant while ZNF281was resistant to β-TrCP2 lacking the F box (Figure 7B). These findings were further confirmed through an *in vitro* ubiquitination assay. As shown in Figure 7C, purified β-TrCP2, but not β-TrCP2 lacking the F box, induced ZNF281 ubiquitination which also required its phosphorylation by GSK-3β. As UV irradiation can activate GSK-3β [19], we then examined whether GSK-3β/β-TrCP2 was a dependent pathway for ZNF281 degradation under pathophysiological condition. The results showed that knockdown of β-TrCP2 and GSK-3β using siRNA blocked the UV-induced ZNF281 degradation (Figure 7D).

Next, we sought to determine whether the GSK-3β–dependent, β-TrCP2–promoted, degradation of ZNF281 affects its transactivational activity. The reporter co-transfection assays indicated that GSK-3β activation plus β-TrCP2 overexpression resulted in a significant suppression of ZNF281 transactivational activity (Figure 7E). ZNF281-S638A abrogated the inhibitory effect of GSK-3β on the activity of ZNF281 whereas Akt-DD (mutations with D(308)T and D(473)S), a constitutively active Akt which inactivates GSK3β by phosphorylating GSK3β, significantly enhanced the activity of ZNF281 (Figure 7E). In contrast to WT-ZNF281, β-TrCP2 overexpression failed to affect inhibition of mutant ZNF281-S638A (Figure 7E). Collectively, these findings showed that β-TrCP2 mediates ubiquitination and degradation of phosphorylated ZNF281 by GSK-3β in CRC cells.

### ZNF281 binds to β-TrCP2 promoter and represses β-TrCP2 transcription in CRC cells

**Figure 8:**
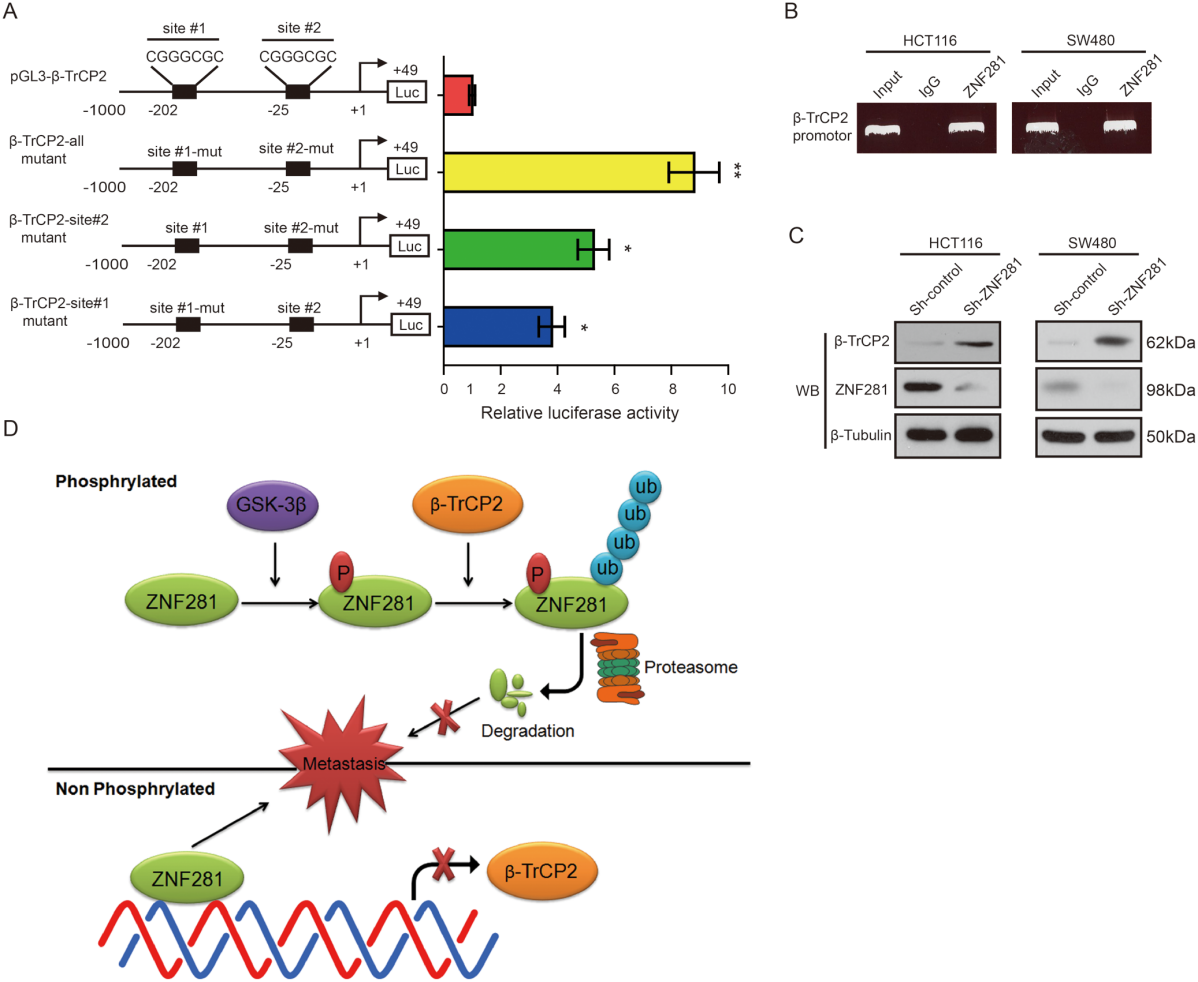
ZNF281 negatively regulated β-TrCP2 transcription in CRC **(A)** Schematic diagram of the β-TrCP2 proximal promoter reporter. The nucleotide positions and sequences of two putative ZNF281 binding sites are shown. Luciferase activity of the indicated pGL3-β-TrCP2 promoter-reporter constructs after transfection into HEK293T cells are shown. **(B)** ChIP assay was performed using a specific anti-ZNF281 antibody. **(C)** CRC cells were transduced with a Sh-control or Sh-ZNF281 for 24 h. And the levels of ZNF281 and β-TrCP2 protein were determined using Western blot analysis. **(D)** Schematic of GSK3β-dependent ZNF281protein ubiquitylation and degradation byβ-TrCP2. *P <0.05, **P <0.01.

Because ZNF281 was a transcriptional factor, which mediates both transcriptional repression and activation of its target genes via binding to GC-rich promoter sequences [8]. Therefore, we wondered whether ZNF281 regulated β-TrCP2 expression. Sequence analysis from position −1000 to +49 relative to the putative transcription start site revealed two putative β-TrCP2 binding sites (GC-rich sequences) from position −25 to −19 and from position −202 to −196 (Figure 8A). Thus, we hypothesized that ZNF281 would regulate transcription of the β-TrCP2 promoter-luciferase reporter. Co-transfection of a ZNF281 vector with the β-TrCP2 promoter-luciferase reporter induced a significant decrease in luciferase activity (Figure 8A). Similar results were also observed in HCT116 cells (data not shown). To determine whether the ZNF281-binding sites within the −25 to −19 and −202 to −196 regions are critical for this regulation, we mutated the ZNF281-binding sites in the β-TrCP2 promoter region. We observed that the co-transfection of the ZNF281 vector with the β-TrCP2-all mutant promoter-luciferase reporters exhibited nearly 8.5-fold increased luciferase activity compared with WT-β-TrCP2 luciferase reporter whereas any single site mutant showed nearly 4-fold increased luciferase activity (Figure 8A). These results suggest that ZNF281 can repress the β-TrCP2 promoter.

ChIP assay indicated that ZNF281 could bind to the β-TrCP2 promoter *in vivo* (Figure 8B). Consistently, decreased ZNF281 expression promoted β-TrCP2 expression in CRC cells (Figure 8C). These results support our hypothesis that ZNF281 binds to the putative region of the β-TrCP2 promoter *in vivo* and represses β-TrCP2 transcription, thus exerting a negative feedback loop.

## DISCUSSION

The regulation and functional analysis of ZNF281 still is in its beginnings. Here, we reveal a novel posttranslational modification of ZNF281 in CRC. We found that GSK-3β-mediated phosphorylation of ZNF281 at Ser638 promoted its ubiquitination by β-TrCP2 and subsequent degradation. Furthermore, we also showed that ZNF281 transcriptionally inhibited the level of β-TrCP2, highlighting the importance of a negative feedback loop in CRC cells.

The current study extends the knowledge on the regulation of ZNF281. Indeed, previous studies reported that the transcription of ZNF281 is upregulated by SOX4 and snail [12], while miR-34a and miR-203 significantly decrease the level of ZNF281 via directly targeting its 3’-UTR [13, 20]. In addition, DNA damage-inducing drugs can increase ZNF281 expression in cancer cell lines [21]. Moreover, the ZNF281 protein is phosphorylated by ATM and ATR kinases upon DNA damage [14]. We provided strong evidence to show that GSK-3β is another new kinase to phosphorylate ZNF281, which subsequently leads to ZNF281 ubiquitination and degradation. Thus, we uncovered a novel mechanism underlying the Ub-mediated degradation of ZNF281 protein in CRC progression. Of note, autophagy is another important protein degradation mechanism in addition to the ubiquitin proteasome system (UPS) pathway. They are not mutually exclusive, but coordinate the regulation of protein degradation. Moreover, autophagy and the proteasome share common substrates as well as regulatory factors depending on particular context [22]. So far, there is no report on whether autophagy affects ZNF281 protein degradation. Therefore, it would be a very interesting research direction. Further studies are needed to elucidate the role of autophagy in ZNF281 protein degradation.

Our findings indicate that β-TrCP2 interacts with ZNF281, which promotes Ub-mediated degradation of ZNF281 protein and inhibits CRC growth and metastasis. This is the first time that β-TrCP2 is shown to regulate ZNF281 ubiquitination and its oncogenic function. Targeting the GSK-3β/β-TrCP2/ZNF281 axis could be exploited for therapeutic purposes in patients with CRC.

Our results indicated that GSK-3β directly phosphorylated ZNF281 at Ser638 site, but we did not rule out the possibility that other serine or threonine on ZNF281 can be phosphorylated by GSK-3β. Although several other conserved GSK-3 motifs also exist on ZNF281, our data indeed demonstrated and highlighted the importance of ZNF281 phosphorylation at Ser638 site. We reasoned that ZNF281 phosphorylation at Ser638 might be due to its unique location to β-TrCP2 degron. Future studies are needed to delineate the significance of other phosphorylation sites of ZNF281. In addition, unlike other substrates of GSK-3β, such as c-Myc and β-catenin [23, 24], we did not identify the priming phosphorylation kinase of ZNF281. And our findings imply that the ZNF281 phosphorylation by GSK-3β might not require priming phosphorylation. However, following the serine 638 of the phosphorylation motif on ZNF281, there are several glutamic acid residues, which may mimic phosphor-serine/threonine, thus act as priming phosphorylation. This hypothesis needs further investigation in the future.

Our report also raises several questions. Firstly, why do just β-TrCP2 and GSK-3β promote the ubiquitination of ZNF281while GSK-3α and β-TrCP1 fail to interact with and posttranslationally modify ZNF281? Secondly, we did not identify which lysine is the site for K48-linked polyubiquitin chains on ZNF281. Thirdly, whether other posttranslational modifications such as acetylation and methylation also regulate the level of ZNF281 in CRC? Importantly, the prognostic value of the correlation between β-TrCP2/GSK-3β and ZNF281 in CRC and other malignancies also needs to be further evaluated. Additional experiments will be needed to answer all of these questions, which will significantly contribute to the understanding of the control of ZNF281 stability in cancer cells and may provide opportunities for developing innovative anticancer therapeutic modalities.

In summary, we provide the prior direct evidence that ZNF281-mediated CRC growth and metastasis can be rescued by GSK-3β and β-TrCP2 overexpression. Mechanistically, GSK-3β-mediated phosphorylation of ZNF281 recruited E3 ligase β-TrCP2 which directly ubiquitinated ZNF281 and induced its degradation in CRC. In turn, ZNF281 also transcriptionally repressed the expression of β-TrCP2, thus forming a negative feedback loop (Figure 8D). Our findings suggest that GSK-3β/β-TrCP2/ZNF281 loop can serve as a potential therapeutic target for CRC patients.

## MATERIALS AND METHODS

### Cells, antibodies, constructs, and reagents

Human 293 T cells and colon cancer cell lines: HCT116, SW620 and SW480 cells were purchased from the American Type Culture Collection (Manassas, VA, USA) and maintained in DMEM (Gibco, Grand Land, NY, USA) supplemented with 10% fetal bovine serum and 1% penicillin/streptomycin 100 μg/ml streptomycin. GSK-3β, β-TrCP2, and β-TrCP1 knockout MEFs were prepared as described previously. Cells were grown at 37°C in a 5% CO2 atmosphere within a humidified incubator. The following antibodies were used: an anti-phospho-Ser antibody (Invitrogen, Carlsbad, CA, USA), anti-GSK-3β (BD Transduction Labs, Chestnut Hill, MA, USA), phospho-Ser9-GSK-3β (Cell Signaling Technology), GSK-3α (Stressgen Biotechnologies), ubiquitin, β-tubulin, and β-TrCP1, β-TrCP2 (Zymed, CA, USA). HA tag, Myc tag and Flag tag antibody were obtained from Sigma (St. Louis, MO, USA). Anti-K48-polyubiquitin antibody (Apu2.07) and anti-K63-linked polyubiquitin antibody (Apu3.A8) were a kind gift from Bing Wang (Rutgers University). PCGN-GSK-3β, pGEX-GSK-3β, and GSK-3β-S9A, pMT2-GSK-3α, β-TrCP1, β-TrCP2, and the F-box-domain-deleted mutant β-TrCP1/ΔF box were purchased from Addgene. All other mutants were generated with the QuickChange multisite-directed mutagenesis kit (Stratagene) according to the manufacturer's protocol, and verified by automated sequencing. GSK inhibitor, Cycloheximide (CHX) and MG132 were from Millipore and Sigma respectively.

### SiRNA, shRNA and transfection

β-TrCP2-small interfering RNA (siRNA) (5′-GAGCUCUUGGUGGAUCAUCTT-3′), control siRNA duplex (Dharmacon, CO), and GSK-3α and GSK-3β siRNA expression plasmids (Upstate Biotechnology) were transfected with Lipofectamine 2000 (Invitrogen, CA) or electroporation using Nucleofector 1 (Amaxa Biosystems, MD). The shRNA lentiviral plasmids (pLKO.1-puro) against human β-TrCP were purchased from Sigma. The sequences targeting β-TrCP1 are 5′-CCGGGCACATAAACTCGTATCTTAACTCGAGTTAAGATACGAGTTTATGTGCTTTTT-3′ and 5′-CCGGGCGTTGTATTCGATTTGATAACTCGAGTTATCAAATCGAATACAACGCTTTTT-3′; the sequences targeting β-TrCP2 are 5′-CCGGAGAAGACTTGGCCTCTAATTTCTCGAGAAATTAGAGGCCAAGTCTTCTTTTTTG-3′ and 5′-CCGGTATCAGTGGCCTACGAGATAACTCGAGTTATCTCGTAGGCCACTGATATTTTTG-3′. HEK 293T cells were transfected with indicated lentiviral vector. 24 hours later, puromycin (1g/ml) was added to the cells to select puromycin-resistant clones.

### *In vitro* kinase assay

In the presence of 50 mM ATP, GST-GSK-3β (Bio-Rad) was mixed with GST-ZNF281 proteins in 20 μl of kinase assay buffer with or without 5 μCi [γ-32P]ATP for 30 min at 30°C. Reaction products were subjected to western blot, analyzed with indicated antibody, or visualized by autoradiography.

### *In vivo* ubiquitination assays

293T cells were transfected with HA-ubiquitin, β-TrCP1/2 or F-box-deleted β-TrCP1/2, and other indicated plasmids, and then treated with MG132 (10 μM) for 10 h. ZNF281 was immunoprecipitated and then blotted with anti-HA-ubiquitin.

### Western blotting

Western blot analysis was performed as previously described [25].

### Co-immunoprecipitation

Based on the manufacturer's instructions, cells were transfected with plasmids with Lipofectamine 2000 (Invitrogen). After 48 h, cells were collected by washing with cold phosphate-buffered saline, and then lysed with immunoprecipitation (IP) buffer (20 mM HEPES [pH 7.6], 0.5% Triton X-100, 150 mM NaCl, protease inhibitor cocktail tablets, 12.5 mM β-glycerophosphate, 10 mM NaF, 1 mM Na3VO4 1.5 mM MgCl2, 2 mM EGTA, 1 mM phenylmethylsulfonyl fluoride [PMSF]). The samples were incubated and then centrifuged at 13,200 rpm for 10 min. The supernatants were incubated with protein A-Sepharose beads and 1 μg the indicated antibody. The precipitates were loaded and subjected to Western blotting with the indicated antibodies.

### Chromatin immunoprecipitation

We used Chromatin immunoprecipitation (ChIP) by SimpleChIP Plus Enzymatic Chromatin IP Kit (Cell Signaling Technology). In brief, cells were treated with 1% formaldehyde to crosslink proteins to DNA for 10 min at room temperature. Then glycine was added to quench. Crosslinked chromatins were digested and 1 μg of antibody per IP was used to immunoprecipitate the crosslinked DNA. The DNA after being reverse crosslinked was purified and eluted into 50 μl of elution buffer. QRT-PCR was used to measure the amount of immunoprecipitated DNA residing in the β-TrCP2 promoter region with primers targeting β-TrCP2 promoter. The primers were as follows: forward 5′-ggtgacttcctatcctcaat-3′ and reverse 5′-cggcctcgatctatggcacg-3′ (site1) and forward 5′-cgacctgcgggaatcgttct-3′ and reverse 5′-gaaggcgagggggagaagga-3′ (site2).

### Transwell migration assays

A Boyden chamber with 8μm-pore filter membrane was used. Briefly, cells (1×105) in culture medium containing free FBS were seeded in the upper chamber, and the culture medium with 20% FBS was added in the lower chamber as a chemoattractant. After incubation for 48 h, the chamber was fixed in 4% paraformaldehyde and stained with Haematoxylin. Cells on the upper side of the filter were removed with cotton swabs. Cells that migrated to the lower side were fixed in 4% paraformaldehyde and stained with Haematoxylin. The migratory cells on the lower surface of the filter were counted (10 random 200×fields per well). Three independent experiments were performed and the data were presented as the mean ± s.d.

### RNA extraction and real-time polymerase chain reaction (RT-PCR)

Total RNA from cell lines homogenized with Trizol (Invitrogen) was extracted with RNA extraction kit (Qiagen). The RNA was reverse transcribed to DNA using the High Capacity cDNA Reverse Transcription kit (Applied Biosystems, Foster City, CA). Expression of ZNF281, Cyclin D1, Vimentin, and E-cadherin mRNA were evaluated by using Power SYBR Green PCR Master Mix (Applied Biosystems). GAPDH mRNA was used as an endogenous control. Expression of RNA was analyzed using the 2-ΔΔCt method. Primers for mRNA expression were as follows. ZNF281-F: 5′-GAGGACACATAGTGGAGAAAAGCC-3′ and ZNF281-R: 5′-GTGGATCTAGACTAGGGCTGGAGTT-3′; GAPDH-F: 5′-ACAACTTTGGTATCGTGGAAGG-3′ and GAPDH-R: 5′- GCCATCACGCCACAGTTTC-3′; cyclin D1-F: 5′-TGCCATCCATGCGGAAA-3′ and cyclin D1-R: 5′-AGCGGGAAGAACTCCTCTTC-3′; E-cadherin-F: 5′-CACCTGGAGAGAGGCCATGT-3′ and E-cadherin-R: 5′-TGGGAAACATGAGCAGCTCT -3′. In addition, the β-TrCP1 and β-TrCP2 knockdown cells were generated by sequential infection. The knockdown efficiency was confirmed by real-time PCR with the following primers: 5′-CAGTACAGGGACAGGCTGGT-3′ and 5′-TACAACGCACCAATTCCTCA-3′ for β-TrCP1 and 5′-AAACCAGCCTGGAATGTTTG-3′ and 5′-CGTGTTCACATCCCACACTC-3′ for β-TrCP2.

### Dual luciferase reporter assay

Cells were co-transfected with wild-type and mutant reporters and the expression vectors pcDNA3-β-TrCP2 using Effectene Transfection Reagent (Qiagen). Luciferase assays were carried out using the Dual Luciferase Reporter assay (Promega). Each experimental point was analyzed in triplicate in three independent experiments.

### Cell counting Kit-8 (CCK-8) assay

Indicated cells were were cultured with fresh medium for 24 h. At specific time points, Cell Counting Kit-8 (CCK8, Dojindo, Japan) was added, and the cells were incubated for another 4 h. The absorbances (optical densities) were recorded with a universal microplate reader (Bio-Tek) at 450 nm. Each experiment was conducted in triplicates.

### Clinical specimens and immunohistochemistry

Sixty formalin-fixed paraffin-embeded CRC and corresponding non-tumor tissues were collected in accordance with a protocol approved by the Ethical Committee of The First Affiliated Hospital of the Harbin Medical University. All patients’ informed consent was obtained. IHC staining was described previously [25]. To semiquantify staining intensity, a scoring method [26] (H-score) was employed with minor modification.

### Animal studies

All procedures were performed in compliance with guidelines of Institutional Animal Care and Use Committee. SICD mice were implanted subcutaneously into the flank with 1×106 indicated CRC cells. Tumors were observed every 3 days and continued to record for the indicated time. Tumors were dissected and weighted at day 28 when they reached to approximately 1 cm in diameter. The tumor sizes were measured once per week with a Vernier caliper. For detecting lung metastases, SCID mice were injected by way of the tail vein with indicated cells. Bioluminescence imaging was performed using the Xenogen IVIS Spectrum Imaging System (Caliper Life Sciences, Hopkinton, MA, USA) after injection i.p. with 150 mg/kg luciferin. Surface nodules were counted. H&E was performed for histological examination.

### Statistical analysis

Statistical analysis was performed with the SPSS for Windows, release 10. All tests were two-tailed (GraphPad Prism Software version 5.04). Data are presented as mean ± SEM and less frequently are presented as mean ± SD. Comparisons between two groups were made using 2-tailed Student’s t test or Fisher’s exact test. Differences among more than two groups were compared using one-way ANOVA. The Kaplan–Meier and log-rank tests were used for the cumulative survival analysis. The significance level was defined as p< 0.05 (*), p<0.01 (**) and ns p>0.05 (#).

## SUPPLEMENTARY MATERIALS TABLE



## Abbreviations

β-TrCP: the β-transducin repeat-containing protein
CCK-8: cell counting Kit-8
ChIP: chromatin immunoprecipitation
CHX: cycloheximide
CRC: colorectal cancer
EMT: epithelial-mesenchymal transition
GSK: glycogen synthase kinase
ZNF281: zinc finger protein 281
