# Supplementary material for: GSK-3β phosphorylation-dependent degradation of ZNF281 by β-TrCP2 suppresses colorectal cancer progression
Source: Oncotarget. 2017 Aug 9;8(51):88599–612. doi: 10.18632/oncotarget.20100 (PMC5687630; doi:10.18632/oncotarget.20100)
Supplement: Supplementary file 1 [file oncotarget-08-88599-s001.pdf]

GSK-3β phosphorylation-dependent degradation of ZNF281 by β-TrCP2 suppresses colorectal cancer progression

SUPPLEMENTARY MATERIALS

Supplementary Table 1: Relationships between β-TrCP2 and ZNF281 expression in CRC

	ZNF281 expresssion		
	**Low**	**High**	**p-value**
β-TrCP2 expression			
Low	5	30	
High	18	7	
			< 0.01

The “Low” and “High” expression of β-TrCP2 and ZNF281 in CRC tissues were determined according to their H-scores compared with controlled non-tumor tissues. Correlation between β-TrCP2 and ZNF281 was analyzed by Fisher’s exact test ($p < 0.01$).
